# Artificial Intelligence and Machine Learning in Chronic Airway Diseases: Focus on Asthma and Chronic Obstructive Pulmonary Disease

**DOI:** 10.7150/ijms.58191

**Published:** 2021-06-01

**Authors:** Yinhe Feng, Yubin Wang, Chunfang Zeng, Hui Mao

**Affiliations:** 1 Department of Respiratory and Critical Care Medicine, West China Hospital, Sichuan University, Chengdu, Sichuan Province, China.; 2 Department of Respiratory and Critical Care Medicine, People's Hospital of Deyang City, Affiliated Hospital of Chengdu College of Medicine, Deyang, Sichuan Province, China.

**Keywords:** artificial intelligence, machine learning, chronic airway diseases, asthma, chronic obstructive pulmonary disease

## Abstract

Chronic airway diseases are characterized by airway inflammation, obstruction, and remodeling and show high prevalence, especially in developing countries. Among them, asthma and chronic obstructive pulmonary disease (COPD) show the highest morbidity and socioeconomic burden worldwide. Although there are extensive guidelines for the prevention, early diagnosis, and rational treatment of these lifelong diseases, their value in precision medicine is very limited. Artificial intelligence (AI) and machine learning (ML) techniques have emerged as effective methods for mining and integrating large-scale, heterogeneous medical data for clinical practice, and several AI and ML methods have recently been applied to asthma and COPD. However, very few methods have significantly contributed to clinical practice. Here, we review four aspects of AI and ML implementation in asthma and COPD to summarize existing knowledge and indicate future steps required for the safe and effective application of AI and ML tools by clinicians.

## Introduction

Recent developments in computer operations and the rapid development of “big data” have significantly advanced artificial intelligence (AI) and machine learning (ML) technology and their applications in various fields such as medicine 1. Medical data are difficult to capture, manage, and process using conventional tools in a timely manner because the datasets are huge, they are frequently updated, and the data come in diverse formats. Instead, imaging, genomic, proteomic and electronic health records (EHRs) data can be mined using AI/ML to extract new knowledge 2. This development has led to rapid changes in the use of AI/ML in medicine, especially in medical imaging 3, where the techniques are used not only for rapid disease screening, but also to improve diagnostic accuracy and work efficiency 4. Genomic data are another enormous source of complex medical information that has recently emerged. Recent studies have demonstrated that the systematic analysis of genomic data with AI/ML technology can favor precision medicine for the benefit of patients 5, 6. Although the most widely used AI/ML technology in respiratory diseases is chest imaging, especially for the screening and diagnosis of lung nodules, the application of AI/ML tools in chronic airway diseases is attracting increasing attention 7, 8.

**Figure 1:**
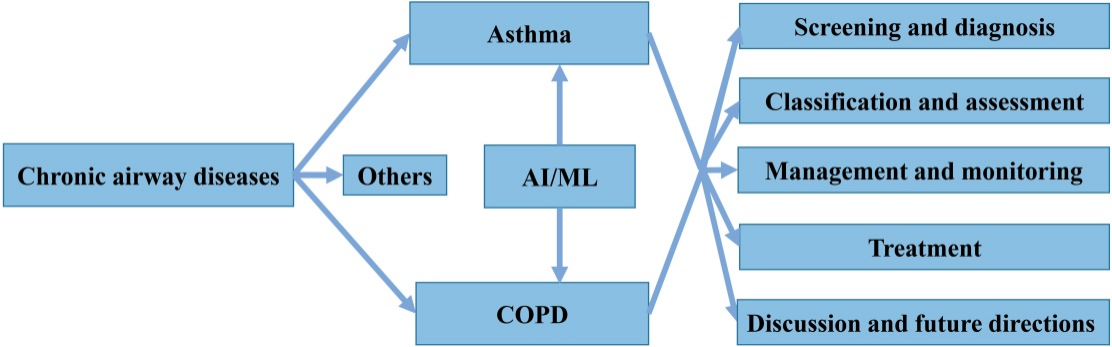
Structure of the present review.

Chronic airway diseases, such as asthma, chronic obstructive pulmonary disease (COPD) and bronchiectasis, are lifelong and life-threatening pathological conditions that extensively affect people of all ages, races, and sex worldwide 9. These diseases are characterized by airway inflammation, obstruction, and remodeling, and common symptoms include cough, sputum, and shortness of breath. Their etiology and pathogenesis are complex and not yet fully understood 10, 11. Patients with chronic airway diseases are also prone to relapse, increasing their risk of hospitalization and death, and seriously affecting their quality of life. Among these diseases, asthma and COPD lead to the highest morbidity and socioeconomic burden worldwide 12. Despite extensive efforts, identifying, treating and managing both disorders still face many challenges, such as under- and overdiagnosis, unclear pathogenesis, lack of uniform classification criteria for phenotypes, and high risk of death and high costs associated with exacerbations 13. In addition, several AI/ML methods have recently been applied for both diseases, but only a few have significantly contributed to clinical practice. Thus, summarizing existing knowledge and indicating future directions is required for the safe and effective application of AI/ML tools by clinicians. Here, we systematically review the application of AI/ML technology to four different aspects of asthma and COPD: screening and diagnosis, classification and assessment, management and monitoring, as well as treatment (**Figure 1**). We also present the development of several models based on ML algorithms.

## General concepts, terminologies and limitations of AI/ML

In order to facilitate understanding, we quickly explain the general concepts and terminologies of AI/ML that commonly appeared in this review. In addition, we summarize the evaluation indicators and current limitations of ML.

**Figure 2:**
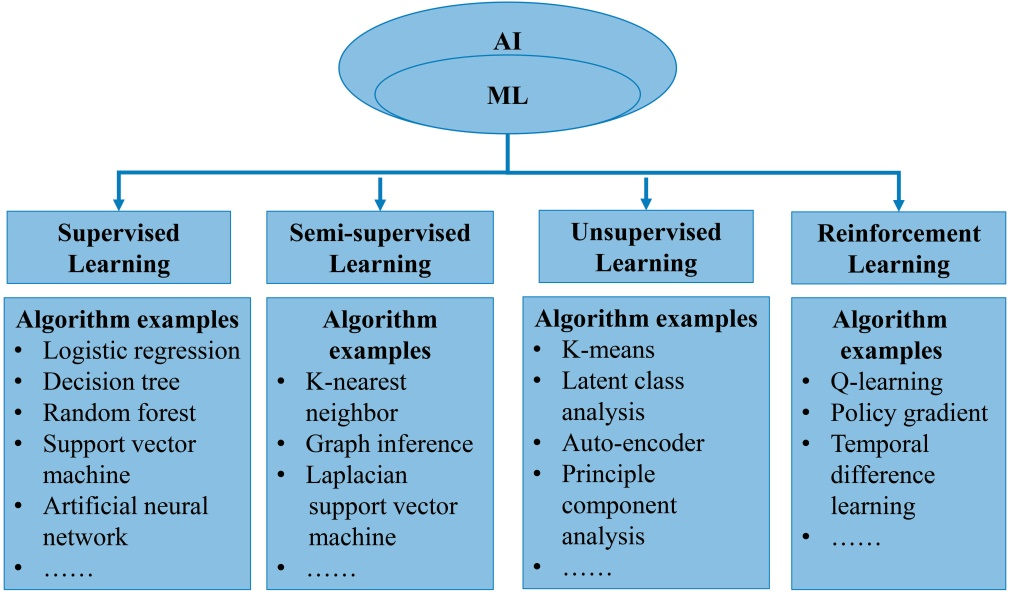
Categories of machine learning algorithms.

**Figure 3:**
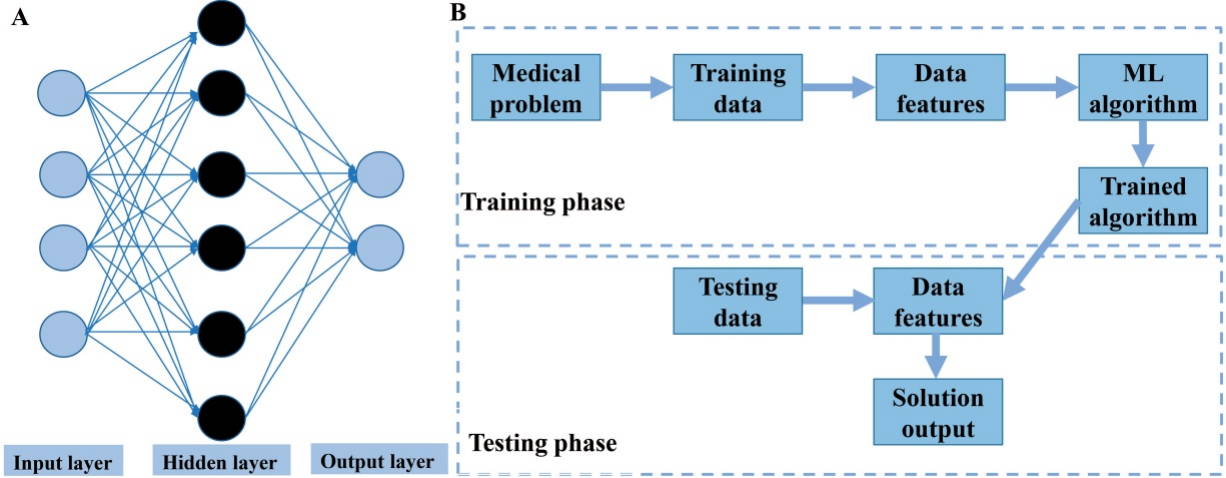
Overview of machine learning. **A.** Illustration of an artificial neural network algorithm. The structure of artificial neural network includes three main layers, namely input layer, hidden layer and output layer. The input layer represents the features extracted from data, which are then integrated by the hidden layer (one or more) to obtain transformed features. Finally, the transformed features are used by the output layer to predict the outcome. **B.** Common paths for training and testing machine learning model in medicine.

In general, AI refers to the technology that represents human intelligence through computer programs. ML is a branch of AI technology based on statistical techniques for self-learning and the development of problem-solving skills. In particular, ML uses complex algorithms to analyze large amounts of data, identify patterns, make predictions that do not require specific codes, and evolve with increasing sample size to improve learning. ML technology can be divided into supervised, semi-supervised, unsupervised and reinforcement learning 14, 15 (**Figure 2**). Supervised learning trains ML algorithms to labeled data. These labels, that include data types, data attributes and feature point locations, are used as expected effects to continuously modify the prediction results of the ML model. Common tasks for supervised learning are regression and classification for continuous and categorical outcome variables, respectively 15. Semi-supervised learning can fit models to not only labeled data but also unlabeled data. When this type of ML algorithm classifies unlabeled data, it usually measures the distance/similarity between the target sample and all labeled samples 16. Unsupervised learning aims to explore and infer potential natural connections and groupings from unlabeled data 17. Reinforcement learning, in contrast, is a general term for ML approaches that integrate prediction and decision-making. This type of ML technology has an iterative learning approach, and can self-adapting according to the initial feedback 17. **Table 1** and** Figure 3A** introduce and quickly explain common ML algorithms reviewed in this article. Based on different ML algorithms, several ML models with different functions have been developed so far. It is important to note that an optimal ML model cannot be easily developed with a limited dataset. Instead, a satisfactory ML model should be constructed in two phases: the model is developed in the training phase and then its performance is assessed in the testing phase 18 (**Figure 3B**).

In order to evaluate the performance of trained ML models, several reasonable evaluation indicators must be used. Generally, the ML model selects different evaluation indicators according to the different classification and regression tasks. In classification, the evaluation indicators are often accuracy, false positive rate, false negative rate, sensitivity (recall), specificity, precision, F1-score, C-index (concordance index), receiver operating characteristic curve and the area underneath it (AUC). The regression tasks focus on the difference between the predicted and true value. Therefore, the evaluation indicators include mean square error, root mean square error, mean absolute error, and median absolute deviation.

Although ML technology is continuously growing in the medical field, its application is greatly limited due to issues related to the availability of adequate data (e.g. text, numbers, images), experiments and methods, and ethics 14, 15. Inaccurate or missing data can cause serious problems, leading to incorrect model structure and biased conclusions. The imbalance and sparsity of categories in medical data can also limit ML application. Therefore, repeated experiments need to be performed and different ML methods should be explored for addressing medical challenges. Experimental design and replication, model selection, model generalization, and model interpretability are crucial aspects of applying ML techniques 19. A good experimental design can reduce experimental errors and give more accurate conclusions. Model selection is one process of finding a solution to the research problem, but there are currently no standards to guard against model misuse or abuse. ML can also improve model generalizability to ensure more accurate prediction of future cases, but how this is best done requires further study. Interpretability of a model makes it more relevant to medical decision making, but most data-driven ML techniques remain unexplored. Another challenge of using AI/ML is to ensure ethics and eliminate prejudice during their application 20. Ethical problems can arise due to problems with optimization, prediction, or classification, which can lead to inequality on sensitive issues or to violations of privacy. Research should not only build ML models but also resolve ethical issues associated with data use and interpretation. Despite these current limitations, AI/ML techniques are needed in the medical field due to the special ability to efficiently analyze and integrate large and heterogeneous data.

## AI/ML and asthma

### Application of AI/ML to asthma screening and diagnosis

As a heterogeneous disease, asthma is often under- or overdiagnosed, especially in poor areas. In fact, almost 20-73% of cases remain undiagnosed, while about 30-35% of people diagnosed with asthma do not actually have the condition 21, 22.

To address this issue, EHRs and Predetermined Asthma Criteria were used in a retrospective birth cohort study to develop for the first time a natural language processing algorithm for pediatric asthma diagnosis with high sensitivity (97%), specificity (95%), as well as positive (90%) and negative (98%) predictive values. The test cohort of this study consisted by 497 children, among whom the asthma prevalence was 31%. The application of the same algorithm to records from 497 children (median age, 2.3 years) at another hospital showed similar sensitivity (92%), specificity (96%), and positive (89%) and negative (97%) predictive values, confirming the algorithm's efficiency in diagnosing pediatric asthma in an external EHR system. However, the algorithm should be further validated on an adult cohort 23, 24 (**Table 2**). In another cross-sectional study, an ML model based on natural language processing algorithm was also developed by mining EHRs to automatically screen pediatric patients who met the Asthma Predictive Index criteria for asthma diagnosis. A total of 427 subjects with an average age of 5.3 years were enrolled in the test phase, and the sensitivity, specificity, and positive and negative predictive values of the ML model reached 86%, 98%, 88%, and 98%, respectively 25. These results suggest that ML models based on natural language processing can be used to identify pediatric patients with undiagnosed asthma. In addition, an artificial neural network model based on 13 clinical characteristics was developed using clinical findings from EHRs, which was able to identify 100% asthma patients among 254 individuals 26.

Although spirometry and bronchial provocation tests are increasingly available, they require the full cooperation of patients and cannot confirm correct diagnosis of asthma. Therefore, the non-invasive forced oscillation technique, which does not require patient cooperation, was combined with four ML algorithms (k-nearest neighbor, random forest, decision trees, and a feature-based dissimilarity space classifier) to produce ML classifiers that serve as a useful and portable tool for diagnosing asthma airway obstruction 27. Among the four algorithms, k-nearest neighbor led to the highest AUC of 0.91. Further research combining the forced oscillation technique with ML algorithms (k-nearest neighbor, random forest, AdaBoost with decision trees, and support vector machine) resulted in several novel classifiers that achieved AUC ≥0.9 for the differential diagnosis of patients with asthma or restrictive respiratory diseases in 97 individuals. However, the results should be further verified on an external dataset 28.

Despite the lack of specific biomarkers for asthma, its diagnosis can be improved by combining multiple methods and clinical data. For instance, a novel AI system (Mahalanobis-Taguch) was developed based on ML algorithm and several biomarkers determined from routine blood samples, such as platelet distribution width, white blood cell count, and eosinophil count. This system was trained using data from 319 asthmatic patients, then validated in 35 asthmatic patients with a classification accuracy of 94.15% 29. Further confirmation of the effectiveness of this AI system in clinical practice will simplify the diagnosis of asthma. In another study, a random forest classifier based on nuclear magnetic resonance spectroscopy of exhaled breath condensate was developed using the metabolome as the biomarker source 30. The classifier differentiated asthma patients and healthy controls with 80% sensitivity and 75% specificity. However, the sample in the study (n = 109) was relatively small, and no actual metabolites were measured, suggesting that the method requires further validation.

A recent systematic review has also suggested that automated analysis of respiratory sounds by ML algorithms can be used for effective screening and diagnosis of respiratory diseases 31. Indeed, multichannel lung respiratory sound signals derived from 30 asthmatic patients and 30 healthy controls were combined with artificial neural network or support vector machine classifiers for the diagnosis of asthma with respective accuracies of 89.2 ± 3.87% and 93.3 ± 3.10% 32. Interestingly, this study did not rely on the presence of the typical wheezing asthmatic symptom as a sound signal. Future studies should collect respiratory sounds of both upper and lower lung lobes for further validation of the results.

Given the usefulness of end-tidal capnography for disease diagnosis, a non-invasive, patient-independent method to process carbon dioxide waveform signals was developed based on the support vector machine classifier to differentiate 30 non-asthmatic and 43 asthmatic patients. The average accuracy, sensitivity, and specificity of the algorithm reached 94.52%, 97.67% and 90%, respectively, suggesting end-tidal capnography as an effective technique for asthma diagnosis 33, 34. However, further validation of the results is required due to the small samples in those studies.

Recently, several classical ML algorithms, such as logistic regression analysis, support vector machine, and deep neural network, were compared for their diagnostic ability when based only on symptoms and physical signs, or when based on the combination of symptoms, physical signs, biochemical findings, lung function tests, and the bronchial provocation tests. That study included 566 adult outpatients and indicated that the deep neural network model was more accurate than other conventional ML tools, reaching an accuracy of 98% when symptoms, physical signs and objective tests were also used 35. This study may be the first to report that AI can perform comparably to human experts for diagnosing asthma in adults. However, the results should be interpreted and generalized carefully, as different ML predictive models perform differently depending on the conditions.

### Application of AI/ML to the classification and assessment of asthma

Asthma is a heterogeneous disease with multiple phenotypes and endotypes that must be properly distinguished for precise prevention and personalized treatment 36-38. In clinical practice, spirometry and bronchial provocation tests are used to assess airflow limitation and hyperresponsiveness, allowing the identification of some asthma phenotypes, while eosinophil count analysis and fractional exhaled nitric oxide measurements can also be applied 39. However, further research is still required to practically and accurately identify the asthma phenotypes.

Latent class analysis can generally fit a probabilistic model to a dataset of several variables such as asthma symptoms or allergy. Therefore, several recent studies have developed, verified, and applied ML-based latent class analysis for asthma classification, indicating its suitability for modelling data from symptomatic or asymptomatic asthma patients 40-44. For instance, based on the data of athlete records that included respiratory symptoms, airway inflammation and hyperresponsiveness, allergic sensitization and lung function test, latent class analysis successfully identified two asthma phenotypes in a total of 150 elite asthmatic athletes who came from Portugal and Norway. The atopic asthma phenotype was defined by allergy symptoms, rhinitis, and high exhaled nitric oxide level, while the sports asthma phenotype was defined by exercise-induced respiratory symptoms and airway hyperresponsiveness, but no allergy 40. The study also found that athletes who practiced water and winter sports were at higher risk of developing the sports asthma phenotype. The validation of this classification method using additional data sources or clinical interventional trials would significantly benefit the personalized treatment of asthmatic athletes.

A predictor pursuit algorithm based on clinical treatment and outcome data was also developed to analyze phenotypes of 1688 childhood asthma patients. Four phenotypes were identified with better (P < 0.001) than traditional ML methods 45. The study also found that nedocromil was better than budesonide in controlling asthma in children with obesity and allergy. A similar classification approach was later reported that focused mainly on the response of severe asthma patients to corticosteroids. Using an unsupervised ML approach (multiple-kernel k-means clustering), four phenotypes were identified in a total of 346 asthma patients. The greatest corticosteroid responsiveness was observed for patients with late-onset, poor lung function as well as high baseline eosinophilia, while the lowest responsiveness was observed for young, obese female patients with severe airflow limitation and mild eosinophilic inflammation 46. Applying these methods to the timely and accurate classification of asthma patients will be a valuable reference for individualized treatment, especially for difficult-to-treat asthma, while reducing the unnecessary use of corticosteroids and related complications.

Precision medicine is an emerging approach of medical science for disease diagnosis and treatment, while genomics is an important manner. In recent years, genetic data have been combined with other clinical information (e.g., demographic, laboratory, and environmental factors) within different ML algorithms to determine asthma phenotypes 47-50. For example, 14 clinical features from 3001 adults with asthma, which included demography, medical history, respiratory symptoms, allergic characteristics, lung function test and bronchial hyperresponsiveness, were integrated with genomics data from previous analyses in order to differentiate asthma phenotypes. Four phenotypes were obtained using latent class analysis algorithm: inactive/mild nonallergic asthma (18%), inactive/mild allergic asthma (37%), active allergic asthma (27%) and active adult-onset nonallergic asthma (18%). This study also identified 15 single nucleotide polymorphisms associated with at least one these four asthma phenotypes, most of them were linked to the “active allergic asthma” phenotype 50. Further research is needed to overcome the limitations of the in-house validation and small sample for genetic analysis, as well as to incorporate more factors and longitudinal data.

ML algorithms have also been used to classify asthma phenotypes according to the disease severity. In particular, latent class analysis was applied to questionnaire data included demographic and clinical features to classify female asthma patients into four phenotypes (“controlled, mild asthma”, “partly controlled, moderate asthma”, “uncontrolled asthma of unknown severity”, and “uncontrolled, severe asthma”) and male asthma patients into three phenotypes (“controlled, mild asthma”, “poorly controlled asthma of unknown severity”, and “partly controlled, severe asthma”) 51. Although the study provided a simpler method for identifying asthmatic phenotypes, there are still several limitations, such as the lack of formal verification of lung function testing. In a similar study, the correlation of wheeze sounds with asthmatic severity was analyzed in 55 asthmatic patients using three ML algorithms, including the ensemble, support vector machine and k-nearest neighbor. The ensemble algorithm showed better performance, and the wheeze sound was identified as a sensitive and specific predictor of asthma severity 52.

### Application of AI/ML to the monitoring and management of asthma

Asthma exacerbation and admission have a significant impact on the life quality and mortality of patients. Artificial neural networks have been extensively used to monitor and manage asthma exacerbation and admission 53-56. For example, an artificial neural network was used to analyze clinical data and create an automated pediatric asthma severity score, which showed better performance than the pediatric asthma score and could therefore help manage pediatric asthma exacerbation in the pediatric intensive care unit 54. Similarly, a retrospective cohort study of 31,433 adult asthma patients reported a time-sensitive predictive model based on an artificial neural network, which integrated clinical variables in the observed time window to predict asthma exacerbation 55. In addition, a modified artificial neural network was applied to predict emergency department visits of asthma and COPD patients due to exacerbation. The developed ML model integrated several daily variables, including the number of emergency department visits as well as meteorological and environmental pollution data, reaching an overall accuracy of 81%. Nevertheless, further studies should include other variables associated with the exacerbation of these diseases 56.

In recent studies, latent class analysis has been used to predict the exacerbation risk of asthma and the decline of lung function in school-age children 57, 58. In the latter study, a dataset consisting of 19 demographic, clinical, and laboratory variables derived from 2,593 children with mild to moderate asthma was used, and the analysis identified allergy and lung function as the main predictors of exacerbation. A similar retrospective cohort study was also performed using EHRs from 2,691 asthmatic children. Among several ML methods, the multivariable logistic regression model proved to be the most accurate, with AUC = 0.86 59. However, all data in that study came from a single medical center, and the multivariable logistic regression model could not be validated.

The application of four ML algorithms (logistic regression, decision tree, naïve Bayes, and perceptron algorithm) to predict severe exacerbations of asthma was recently reported based on daily monitoring data of 576 severe exacerbation events in 2,010 asthma patients. The logistic regression-based model yielded an optimal AUC of 0.85, sensitivity of 90% and specificity of 83% 60. Given the close correlation between severe exacerbations and asthma mortality, the model may be useful to physicians as a reference, but the research data were collected from paper diaries that may be inaccurate.

In order to assess the predictability of an Internet search index for asthma admission, an ML-based prediction model (XGBoost) was developed by combining search index and data, such as air pollution, weather, and previous admission events, yielding a maximum AUC of 0.832. However, the model performance should be further validated in other geographical regions 61. In a similar approach, an artificial neural network model was applied to predict, in real time, asthma-related emergency department visits using environmental and social media data, such Google searches and Twitter 62. The model accuracy was 70%, making it suitable for early intervention in asthma patients to avoid exacerbation. A growing number of studies have also used electronic devices to manage, monitor, and follow asthma patients in real time 63-65. For example, several ML algorithms (naïve Bayesian classifier, adaptive Bayesian network, and support vector machine) were used to analyze telemonitoring data from laptops at home in order to predict asthma exacerbation in a timely fashion. The sensitivity, specificity, and accuracy of the adaptive Bayesian network model reached 100%, but the study was limited by the low number of exacerbations in the collected data 64. Another study used a contactless bed sensor to capture physiological and environmental data for the early detection of asthma exacerbation in children. Using a random forest classification model, an accuracy of 87.4% was achieved 65.

Moreover, a comorbidity portfolio model was also designed based on ML algorithms to improve the prediction of asthma treatment costs over traditional approaches 66. The study found that a combination of cardiovascular and other respiratory diseases was a major risk for increased treatment costs in asthmatic patients. This study provides an important perspective on controlling asthma expenses in light of the high financial burden of asthma worldwide and continuing concern about treatment costs.

Successful monitoring of asthma control levels also plays a significant role in the treatment of the disease. Therefore, physicians' expertise was combined with an ensemble ML algorithm to detect asthma control. The optimal accuracy of the model was 91.66% and, although the study included relatively few factors affecting asthma control, the model could help clinicians develop timely treatment plans 67. Based on these findings, several common supervised ML algorithms were further used to analyze asthmatic monitoring data from 5,875 patients enrolled in the Asthma Mobile Health Study. Both logistic regression and naïve Bayes-based classifiers identified the control level with high accuracy (AUC > 0.87) 68, suggesting that this method could serve as a valuable reference for the treatment of asthma in clinical practice. However, these models should be further validated using more diverse data, preferably data based on objective measures rather than self-report.

### Application of AI/ML to the treatment of asthma

Despite the wide variety of studies on AI/ML implementation in asthma, very few studies have reported the application of AI/ML systems to the treatment of the disease, as such treatment is usually controlled by specific guidelines. In addition to the two aforementioned studies 45, 46, the effects of anti-inflammatory and antioxidative saffron on the treatment of mild-to-moderate allergic asthma in 80 patients were predicted using a genetic algorithm developed by modifying an artificial neural network system. The accuracy of the prediction system was greater than 99% in both the training and testing phase 69, which probably makes it suitable for predicting the treatment effect of other asthma drugs. Nevertheless, the performance of this prediction system needs to be confirmed with studies on more patients with allergic or other types of asthma.

### Discussion and future directions

With the continuous improvement of computer learning and the accumulation of asthma-related data, the application of AI/ML in asthma has made great progress with good results for specific clinical research purposes 70. Using AI/ML techniques, the mining and analysis of huge clinical, metabolomics, genomics, and other heterogeneous asthma data can help to better understand the pathogenesis and guide individual treatment of the disease. Nevertheless, some AI/ML models developed for asthma diagnosis, classification, assessment, and prediction have many limitations, such as a single data source, small sample, or lack of external confirmation, which should be overcome in future studies.

The application of AI/ML in asthma is also still limited. For example, although the current studies can be combined with different data or variables to build ML models for identifying asthma phenotypes, the integration of relative comprehensive data or variables, including demographic, environmental, medical history, symptoms, laboratory examination, pulmonary function testing, genomics, metabolomic, and imaging, is still absent. Moreover, AI/ML methods have rarely been used to identify or predict patients with severe asthma. Such studies would be of great clinical significance, because an in-depth understanding of asthma phenotypes and the reliable identification of specific subgroups could guide the use of specific drugs, such as biological-targeted agents, to bring precision medicine to asthma patients. In addition, most studies on the application of AI/ML in asthma are based on populations and datasets from developed countries, while very little research has been conducted on populations and data in developing countries, where the need for diagnosis, treatment and management is more urgent due to the weaker healthcare systems. To make the situation more challenging, the COVID-19 pandemic has severely disrupted health services for patients with chronic airway diseases. Therefore, the use of AI/ML tools to establish a management system for such patients during an infectious disease epidemic should be seriously considered.

## AI/ML and COPD

### Application of AI/ML to the screening and diagnosis of COPD

There are no specific symptoms related to COPD, but the disease can be diagnosed using the pulmonary function test. However, its accuracy is highly dependent on the patient's cooperation, which explains the common under- and overdiagnosis of COPD in clinical practice 71. To address this challenge, several AI/ML techniques have been used to develop an economical, safe, and effective method for COPD diagnosis. For instance, as an AI diagnostic tool, an “expert system” was built using the following steps: questionnaire formation, WebFlex code development, expert panel pilot validation and clinical validation. The questionnaire information included demography, symptoms, environment, and diagnostic tests. In the clinical validation phase, this “expert system” reached an overall accuracy of 97.5% in 241 patients 72. In a similar manner, a subsequent study used data from lung function tests and clinical information from 1,430 subjects to build AI-based software for the diagnosis of COPD 73. That study showed that the developed software can reach an accuracy of 82% in 50 COPD patients significantly exceeding the diagnostic performance of pulmonologists (44.6 ± 8.7%). It is therefore clear that AI technology can considerably help clinicians make diagnostic decisions for COPD patients.

To reduce the dependence on lung function tests for early diagnosis of COPD, ML has also been used to mine and analyze transcriptomic data extracted from human bronchial epithelial cells, leading to the identification of abnormal expression of 15 genes in the disease, 10 of which had not previously been reported as COPD biomarkers. The different gene combinations were then analyzed by the random forest algorithm to distinguish non-smokers from smokers and COPD patients 74 (**Table 3**). Despite the remarkable diagnostic accuracy of each subgroup (65%), further studies are required to improve the model performance in distinguishing COPD patients from smokers without COPD. Given the lack of specific biomarkers for COPD diagnosis, support vector machine was also integrated with two blood biomarkers, *N*-acetyl-glycoprotein and lipoprotein, which were obtained by comparing 54 COPD patients with 74 normal individuals. The model achieved a diagnostic accuracy of 84.62% and an AUC of 0.90 75, suggesting that the combination of ML algorithms with biomarkers may favor COPD diagnosis and reduce the dependence on lung function tests. However, further validation in a larger patient sample is needed.

Respiratory sounds are an important sign of the lungs and their analysis can be useful in the diagnosis of respiratory diseases 76, 77. In a recent study, 39 features of respiratory sound were integrated with three lung function features derived from 30 COPD patients and 25 healthy subjects, and five ML classifiers were used to categorize normal individuals and COPD patients. Support vector machine and logistic regression achieved a diagnostic accuracy, sensitivity, and specificity of almost 100% 78. In a similar approach, 22 different clinical features were extracted from each of 132 subjects. Based on this dataset, a decision support system was developed to diagnose COPD and asthma, with the random forest classifier showing the highest COPD diagnostic accuracy (97.7%) compared to other techniques. Moreover, smoking, forced expiratory volume in 1 second (FEV1), age, and forced vital capacity proved to be the main predictors 79. However, the results of these studies should be carefully interpreted due to their small, single-center samples.

Inequities in access to medical resources also affect the diagnosis of COPD, especially in less developed areas. Therefore, an automated telehealth AI system was recently developed and verified in 780 patients from several medical institutions 80. The diagnostic accuracy reached 97%, and the simple equipment involved may allow its use in remote areas and in patients with less mobility.

Some patients may also have both asthma and COPD, known as asthma-COPD overlap (ACO). However, the lack of accurate diagnostic criteria has led to insufficient data on the prevalence and treatment of ACO 81. The only relevant study of which we are aware has been registered so far as a protocol, and it aims to precisely classify COPD, asthma, and ACO patients by applying a modified latent class model to EHRs from the Secure Anonymized Information Linkage databank 82. The analyzed data will include demographic characteristics, history of present illness, allergy, and smoking history, so the future study is expected to provide useful information.

### Application of AI/ML to the classification and assessment of COPD

According to the Global Obstructive Lung Disease Initiative, COPD patients are classified into four phenotypes based on their symptomatic assessment, exacerbation and hospitalization history 83. However, the discriminatory ability of this method is insufficient, leading to the AI/ML-based integration of additional information, including physiological features, lung function test results, comorbidities, genome, and biomarkers, for precise phenotype classification, severity assessment, and therapeutic guidance 84-89. For example, k-means clustering was applied to analyze eight factors in 1,195 COPD patients such as physiological features, medical history, COPD assessment test score, and post-bronchodilator FEV1. Four phenotypes were identified: putative asthma-COPD overlap (cluster 1), mild COPD (cluster 2), moderate COPD (cluster 3), and severe COPD (cluster 4). Cluster 4 showed the worst post-bronchodilator FEV1 (46.7%), the shortest 6-min walking distance (365 m), and the highest COPD assessment test score (17.5), whereas cluster 1 showed the highest risk of acute exacerbation 86. Nevertheless, the results need to be supported by a longer follow-up duration (>6 months). In another study, the variation in lung function and life quality scores among 1,676 Asian COPD patients were monitored for one year, identifying three phenotypes of COPD patients. Cluster 1 was defined by worse lung function but fewer symptoms, while cluster 3 showed mild severity but higher body mass index; cluster 2 showed severe disease and more symptoms, including the highest risk of acute exacerbation and rate of FEV1 deterioration 87. However, one of the main study limitations was the high proportion of male subjects (90%). Moreover, using two ML methods (k-means and hierarchical clustering) and based on comorbidities and risk factors, 30,961 COPD patients were classified into five phenotypes: anxiety and depression, severe airflow limitation and weakness, cardiovascular disease and diabetes, as well as obesity/atopy and non-comorbidity 84. Although the aforementioned studies used different ML algorithms and clinical variables and had some limitations, all supported the idea that exploring different phenotypic classification can improve individualized treatment. For similar purpose, the spirometry data of 8980 individuals (COPDGene cohort study) was used to develop a deep neural network model for the identification of four chest computed tomography imaging phenotypes (normal, airway predominant, emphysema predominant, and mixed emphysema/airway). The deep neural network model had a higher accuracy both in the classification of predominant emphysema/airway phenotypes (AUC = 0.80) and predominant emphysema/small airway phenotypes (AUC = 0.91) than FEV1/forced vital capacity, FEV1% predicted and random forest classifier. However, non-smokers with and at risk for COPD should be included in future studies 90.

The assessment of persistent airflow limitation in COPD patients depends on lung function tests. However, only some COPD patients complete these tests in clinical practice, limiting the diagnosis of airway limitation to 56% 91, 92. Considering that it is difficult to identify FEV1 values in structured EHRs, an automatic AI tool was designed to mine FEV1 values in EHRs of 41,689 veterans with COPD. The novel AI tool showed an accuracy of 95%, serving as a helpful tool for the assessment of COPD severity in large patient population 93.

Chest computed tomography has also been widely used to detect lung texture abnormalities and assess the state of COPD. However, a large amount of image data cannot be identified with the naked eye, highlighting the need for AI/ML systems in this field 94. In a recent prospective study, the pulmonary ventilation function of COPD was assessed using a support vector machine and logistic regression algorithms to analyze chest computed tomography images. The assessment model (quadratic support vector machine) was based on 87 image features, and its validity was tested in 27 COPD patients with an accuracy of 88% and an AUC value of 0.82 95. While these results are encouraging, the sample was small and most patients had moderate to severe COPD, suggesting that condign mild COPD patients should be included in future works. In another study, a convolutional neural network algorithm was used to analyze chest computed tomography images from smokers and to assess the diagnosis, stage, exacerbation, and mortality of COPD patients. Smokers in the study were divided into a training phase consisting of 8,983 participants and an evaluation phase consisting of 1,672 participants, which came from COPDGene and ECLIPSE cohorts. The algorithm yielded a c-index of 0.856 for COPD detection and an accuracy of 51.1% for the exact determination of the COPD stage in the COPDGene cohort. Moreover, the c-indices for predicting exacerbation and mortality were 0.64 and 0.72 in the COPDGene cohort, and 0.55 and 0.60 in the ECLIPSE cohort 96. These results indicated better performance of the convolutional neural network in the COPDGene cohort, while suggesting its applicability in stage classification and risk assessment of COPD at the population level. However, this method may have limited applicability because it requires extensive training and computational resources.

Assessing the severity of hospitalized acute exacerbations of COPD (AECOPD) patients is also beneficial to clinical practice. Hence, a modified decision tree algorithm was used to analyze 28 clinical features, including demographics, medical history, and biomarkers derived from 202 inpatients with severe AECOPD and 208 inpatients with mild AECOPD. The classification of severe and mild patients was based on their admission to the intensive care unit. The overall accuracy of the developed classifier reached 80.3%, suggesting that it can be used to assess the severity of hospitalized AECOPD patients 97; however, the patient's body mass index and other inflammatory cytokines should be included in a future prospective study.

### Application of AI/ML to the management and monitoring of COPD

Persistent chronic airway inflammation and airflow limitation in COPD can induce the recurrence of acute exacerbation and readmission. In order to effectively manage COPD patients and monitor the disease, several studies have used ML-based approaches, which proved to be more effective than conventional methods 98-100. In particular, ML algorithms, such as lasso regression and deep neural network, were used to analyze 44,929 COPD hospitalizations divided into a training (70%) and a test (30%) set. The developed models aimed to predict readmission within 30 days after discharge and showed higher prediction ability (c-statistic = 0.61) than the traditional method 98. Similarly, several non-deep and deep ML algorithms were used to mine a database containing medical claims data of COPD patients in order to predict readmission 30 days after discharge, and the optimal AUC was 0.653 99. A retrospective study in France applied decision tree analysis to predict the readmission of 143,006 COPD patients older than 40 years. The study not only showed that the most relevant risk factor of readmission was the number of previous admissions, but it also assessed the cost of readmission within six months 100. Although these studies have reported several limitations, such as the lack of important clinical features, the prediction models could be used by clinicians as a reference.

Persistent airflow limitation along with persistent respiratory symptoms make COPD a lifelong and life-threatening disease. Thus, it is particularly important to monitor variation in lung function and prevent persistent airflow limitation. A ML model based on random forest was recently developed using spirometry data obtained from 4,167 participants in order to predict individuals most likely to develop or have COPD. The primary outcome of the model was FEV1, while the secondary outcome was the risk of airflow limitation (FEV1/forced vital capacity). This model may be a useful tool for personalized risk prediction of airflow limitation and early prevention of COPD 101.

Given the irreversibility of COPD, its early detection and diagnosis are crucial. Thus, six ML models were used to predict the development of COPD based on 101 single-nucleotide polymorphisms and 5 clinical characteristics of 441 patients and 192 normal participants. Among them, 9 single-nucleotide polymorphisms were significantly associated with this disease, including 6 risk and 3 protective factors. In the test set, among the examined models, the k-nearest neighbor classifier and logistic regression showed the highest precision of 82% and accuracy of 81%, while the highest sensitivity (recall) of 100% was achieved using the multilayer perceptron classifier based on the artificial neural network algorithm 102. Although only a few genes and clinical features were included, this model may be effective for early diagnosis of COPD, compensating for the lack of lung function testing among patients in the early disease stages.

ML algorithms were also used to analyze functional respiratory imaging for the prediction of exacerbation and early identification of AECOPD patients 103. Similarly, a series of ML algorithms (logistic regression, random forest, naïve Bayesian, support vector machine and k-nearest neighbor) were used to mine EHRs data derived from 135 AECOPD and 168 control subjects. Further validation and comparison of the developed models indicated that the support vector machine algorithm showed the best performance (AUC = 0.90) 104. Consequently, ML models, and especially the support vector machine model, can help physicians identify AECOPD patients and make timely decisions; however, the models' performance should be further validated using data from external sources. In another approach, a mobile telehealth system was designed to improve self-management in COPD and detect acute exacerbations of stable COPD patients in a timely manner. The system could continuously monitor the clinical information of the enrolled patients at home and warn of an acute exacerbation three days in advance 105. Although the accuracy was only 40% and the study lasted only six months, the development of a simple, effective AI-based monitoring and warning system deserves further investigation.

The global economic burden of COPD increases every year, a trend exacerbated by the aging population. In Europe, the total cost of COPD is estimated at 56% of annual healthcare expenditure for respiratory diseases 106. To identify and predict the costs of COPD patients in China and to provide crucial health management information, three ML algorithms (logistic regression, random forest, and extreme gradient boosting) were used to analyze 54 different demographic parameters and medical information from 780,295 hospitalizations. Although all ML models showed excellent predictive efficiency, the extreme gradient boosting model showed the highest sensitivity (71.3%) and AUC (0.801) 107, indicating that it may serve as a valuable predictive tool for patients, clinicians, insurance policy makers, and other healthcare professionals in developing countries.

Since COPD is one leading causes of death worldwide, some studies have also used AI/ML technology to predict the risk of mortality in patients with COPD 108, 109. For instance, a total of 30 clinical, lung function, and chest imaging features obtained from 3,900 participants with moderate to severe COPD were analyzed to build a random forest model for mortality prediction. The novel model showed good prediction performance (C-index > 0.7), and the optimal risk predictors were the 6-min walk test, the FEV1 value, and the pulmonary artery-to-aorta ratio 109. Hence, the novel ML model can be a useful tool to guide the early intervention of COPD to avoid further deterioration, but more external population cohorts are needed for validation.

### Application of AI/ML in COPD treatment

AI/ML technologies can monitor, integrate, and analyze large-scale, heterogeneous clinical information from COPD patients; suggest optimal individualized treatments; and reduce over- or undertreatment caused by clinician errors. However, similar to asthma, we found only one study related to the application of AI/ML in COPD treatment. In that work, several ML models (e.g. sparse maximum-margin classifier, ensembles of boosted classifier, multitask neural network model) were developed based on 153 predictive factors derived from telemonitoring of physiological, symptom, and baseline data from 135 patients with moderate to severe COPD. The data included demography, severity, quality of life and hospital admissions, and the goal was to detect acute exacerbations and guide the corticosteroid therapy of COPD. Irrespective of acute exacerbations or corticosteroid use, the optimal ML model (multitask neural network) showed better AUC values than non-ML methods (0.74-0.77 vs 0.60-0.66), and its performance was not improved by adding weather data 110. However, the evaluation of model performance relied on cross-validation rather than multiple independent cohorts, suggesting the need for further study.

### Discussion and future directions

Given the structured data from pulmonary function testing and their important role in the diagnosis and management of COPD, AI/ML techniques were combined with such testing early in the field in order to help clinicians assess lung function 111. Other studies have focused on the use of AI/ML methods to reduce the reliance on pulmonary function testing in clinical practice. Our literature review also showed that ML methods have been increasingly used in recent years for the identification of COPD phenotypes and for the prediction of acute exacerbation and death risk. However, similar to the limitations of AI/ML techniques in asthma, such as single-source populations and lack of external validation, the model still needs to be validated in more prospective studies using a large real-world dataset as well as clinical data from continuous monitoring.

Although there have been significant advances in understanding and managing COPD using AI/ML methods, there are still many unmet needs. For example, airflow limitation is the hallmark of COPD, and studies have found that even in patients with similar FEV1 levels, there are significant differences in the degree of respiratory symptoms, frequency of acute exacerbations, and activity endurance 112. The underlying biological mechanism remains unknown, indicating the importance of phenotypic studies in COPD patients. The current four phenotypes of COPD proposed by the Global Obstructive Lung Disease Initiative are insufficient for individualized therapy; thus, the development of phenotype-specific therapeutic strategies should be extensively studied in the future 85. We expect that the integration and analysis of different data using AI/ML technologies may provide new insights into the COPD phenotype.

AI/ML may also play a significant role in the treatment and management of patients with stable chronic airway diseases. Inhalation therapy is the basic therapeutic approach for stable COPD. However, stable COPD patients may misuse inhaled devices due to the diversity of inhaled drugs and devices and the lack of medical assistance. Therefore, the application of AI/ML methods for the early detection of errors and the improvement of patient compliance should be further investigated. AI/ML studies are also worth conducting in low-income individuals or regions, as they usually exhibit high morbidity and experience a heavy financial burden due to COPD; in addition, the relatively delayed generation of clinical data in their health systems leads to poor data integrity.

## Conclusions

In recent years, the application of AI/ML techniques in the medical field has evolved rapidly, while several AI/ML tools have been extensively studied and applied to chronic airway diseases. This review summarizes the recent advances in the implementation of AI/ML techniques in the screening and diagnosis, classification and assessment, management and monitoring, as well as treatment of asthma and COPD. Our research supports that AI/ML techniques can be used effectively to analyze and integrate large, heterogeneous medical data, thus assisting physicians in making decisions and guiding clinical practice. In addition, these techniques can be applied to analyze different responses to treatment, providing therapeutic guidance for specific phenotypes required in precision medicine, and establish a management system for chronic airway disease patients during an infectious disease epidemic. However, the results about AI/ML tools should be interpreted and generalized with caution. The AI/ML techniques cannot yet replace clinicians in the diagnosis and treatment of chronic airway diseases, and further studies are needed to examine the effect of several parameters on ML model construction and to verify existing findings with larger samples and external data sources.

## Figures and Tables

**Table 1:** Summary of common machine learning algorithms

Type of machine learning algorithm	Description	References describing applications
Natural language processing	Natural language processing is a general term for a series of technical methods. It can be divided into natural language understanding (NLU) and natural language generation (NLG). NLU focuses on how to understand text, while NLG focuses on how to generate natural text after understanding the text.	23-25
K nearest neighbor	K nearest neighbor is a type of instance-based learning algorithm, and the training process simply memorize the training data. It categorizes the sample according to the similarity. The similarity is calculated using measures such as Euclidean distance and Hamming distance.	27,28,52, 67,78,79, 94,102,104
Random forest	Random forest is an ensemble learning method. It contains multiple decision trees and integrates these decision trees to category of data. The size of trees and the number of variables usually determine the performance of model.	27,28,30, 47,48,59, 65-67,74,79, 90,99,101, 104,107,109
Support vector machine	Support vector machine is usually used for classification and regression. It learns the optimal hyperplane to classify data. Generally, it has low misclassification error and scale well to high-dimensional data. However, selecting the optimal kernel function is essential.	28,32,35, 52,59,66-68, 75,78,79, 95,99,102, 103,104,108
Artificial neural network	This is a kind of hierarchical nonlinear mapping network based on neurons and activation functions. Its structure includes three main parts, namely input layer, hidden layer and output layer. This structure is used to analyze variables in order to predict an outcome. The primary limitation is the underlying model's lack of transparency.	32,53-56, 62,69,79, 94,102
Latent class analysis	Latent class analysis is a statistically principled technique that is used in factor analysis, cluster analysis, and regression. It is to explain and estimate the association between manifest indicators by latent class variables. This method suits to classify subgroups in large and heterogeneous data.	40-44,50, 51,82
K-means	This method divides the dataset into K clusters, and each cluster is represented by the average value of all samples in the cluster, which is called the "centroid". K-means clustering is easy to interpret and computationally efficient. However, the number of clusters needs to be prespecified.	46,84,86, 88
Logistic regression	Logistic regression estimates the probability of a binary classification problem. The dependent variable of it obeys the Bernoulli distribution, and nonlinear factors are introduced through the Sigmoid function.	47,59,60, 66-68,78,79, 95,99,102, 104,107,108
Decision tree	Decision tree creates a series of decision rules to predict categorical and continuous outcomes based on input variables. It contains three main parts: a root node, leaf nodes and branches. Decision tree is easy to understanding, but unstable and prone to overfitting.	47,49,60, 67,68,78, 79,97,100, 102
Lasso regression	Lasso regression is a linear regression method using L1-regularization. L1-regularization can compress the coefficients of variables and change some coefficients to zero, so as to achieve the purpose of variable selection.	48,59,98
Naïve Bayes	Naïve Bayes is a classification algorithm based on Bayes' theorem, which is suitable for scenarios where variables are independent of each other. It is relatively simple and has good performance in the presence of noise, missing data, and irrelevant variables.	64,67,68, 79,104

**Table 2:** Machine learning studies on asthma

Reference	Category	Study population	ML algorithms	Input features	Studied outcome	Results	Critical appraisal of the study
Wi CI, 2017 23	Screening and diagnosis	927 children: training cohort = 430 test cohort = 497	NLP	Clinical (EMRs)	Pediatric asthmatic subjects or not	Sensitivity = 97%, specificity = 95%, positive predictive value = 90%, negative predictive value = 98%	Pros: use of electronic medical records Cons: single internal electronic medical record system
Wi CI, 2018 24	Screening and diagnosis	595 children: training cohort = 298 test cohort = 297	NLP	Clinical (EHR)	Pediatric asthmatic subjects or not	Sensitivity = 92%, specificity = 96%, positive predictive value = 89%, negative predictive value = 97%	Pros: use of an external electronic medical records system Cons: has not yet been validated on an adult cohort
Kaur H, 2018 25	Screening and diagnosis	514 children: training cohort = 87 test cohort = 427	NLP	Clinical (EHR)	Pediatric asthmatic subjects or not	Sensitivity = 86%, specificity = 98%, positive predictive value = 88%, negative predictive value = 98%	Pros: development of the first algorithm to automatically extract patients who meet the Asthma Predictive Index criteria Cons: relatively small sample
Alizadeh B, 2015 26	Screening and diagnosis	254 subjects: training cohort = 70% test cohort = 30%	ANN	Clinical	Asthmatic subjects or not	Accuracy = 100%	Pros: based on 13 clinical characteristics used by physicians to diagnose asthma Cons: single data source and relatively small sample
Amaral J, 2017 27	Screening and diagnosis	75 stable asthma patients: 39 with airway obstruction and 36 without	KNN, RF, ADAB, FDSC	Forced oscillation technique parameters	Airway obstruction	KNN reached the highest accuracy range (AUC = 0.91)	Pros: use of the non-invasive forced oscillation technique Cons: the exact sensitivity and specificity values are unknown
Amaral J, 2020 28	Screening and diagnosis	97 individuals: controls = 20 asthmatic patients = 38 restrictive patients = 39	KNN, RF, ADAB, SVM	Forced oscillation technique parameters	Asthmatic or restrictive respiratory diseases subjects	All classifiers achieved high accuracy (AUC≥0.9)	Pros: differential diagnosis of asthma and restrictive respiratory diseases Cons: single practice site and relatively small sample
Zhan J, 2020 29	Screening and diagnosis	355 asthma patients and 1,480 healthy individuals	Mahalanobis-Taguchi system	Routine blood biomarkers	Asthmatic subjects or not	Accuracy = 94.15% in asthma patients and 97.20% in healthy individuals	Pros: diagnosis of asthma based on routine blood biomarkers Cons: a complete blood reference space is required to more accurately identify asthma patients
Sinha A, 2017 30	Screening and diagnosis	89 asthmatic subjects and 20 healthy controls	RF	Nuclear magnetic resonance spectra of exhaled breath condensate	Asthmatic subjects or not	Sensitivity = 80%, specificity = 75%	Pros: advocated the use of exhaled breath condensate spectral signatures Cons: did not actually measure any metabolites
Islam MA, 2018 32	Screening and diagnosis	60 subjects: normal = 30 asthma patients = 30	ANN, SVM	Clinical (lung sounds)	Normal or asthmatic subjects	Accuracy = 89.2(±3.87)% in ANN and 93.3(±3.10)% in SVM	Pros: used lung respiratory sound signals Cons: did not collect respiratory sounds of both upper and lower lung
Singh OP, 2018 34	Screening and diagnosis	non-asthmatic = 30 asthmatic = 43	SVM, KNN, NB	Respired carbon dioxide waveform	Asthmatic subjects or not	Accuracy = 94.52%, sensitivity = 97.67%, and specificity = 90% in SVM	Pros: non-invasive, patient-independent method based on simple signal processing algorithm to screen for asthma Cons: relatively small sample
Tomita K, 2019 35	Screening and diagnosis	566 adult out-patients (367 asthma patients)	SVM, DNN	Clinical, Lung function test, Bronchial challenge test	Adult asthmatic subjects or not	Accuracy = 98% in DNN and 82% in SVM	Pros: models based on symptoms, physical signs and objective tests Cons: single practice site
Couto M, 2015 40	Classification and assessment	asthmatic athletes = 150 healthy athletes = 129 athletes with other pathologic conditions = 45	LCA	Clinical (athletes' records)	Asthmatic phenotypes	Two phenotypes: atopic asthma and sports asthma	Pros: identification of asthmatic athlete phenotypes Cons: need to be validated by larger clinical interventional trials
Chen Q, 2012 41	Classification and assessment	689 asthma children	LCA, BIC	Clinical (questionnaire data)	Asthmatic phenotypes	Four phenotypes: never/infrequent, early-transient, early-persistent, and late-onset	Pros: identification of phenotypes based on wheeze Cons: some children could not provide precision data
Weinmayr G, 2013 42	Classification and assessment	>4,000 asthma children	LCA, BIC	Clinical (questionnaire), Bronchial hyperresponsiveness	Childhood asthma phenotypes	Seven phenotypes: one corresponding to healthy children; three related to wheeze; three related to congestion and coughed-up phlegm	Pros: identification of phenotypes according to respiratory symptoms Cons: recall bias
Bochenek G, 2014 43	Classification and assessment	201 aspirin-exacerbated respiratory disease patients	LCA	Clinical (questionnaire, spirometry, blood eosinophilia, urinary LTE4 concentrations)	Subphenotypes within AERD phenotype	Four subphenotypes: asthma with a moderate course; asthma with a mild course; asthma with a severe course; poorly controlled asthma with frequent and severe exacerbations	Pros: identification of aspirin-exacerbated respiratory disease phenotypes Cons: LCA stability over time not established
Havstad S, 2014 44	Classification and assessment	594 asthma children (2 years old)	LCA	Serum IgE data on 10 allergens	Atopic asthma phenotypes	Four phenotypes: low to no sensitization; highly sensitized; milk and egg dominated; peanut and inhalant(s)/no milk	Pros: examination of a more recently born, younger, and racially mixed cohort Cons: lack of additional information on lung function, cytokines, and eosinophils
Ross MK, 2018 45	Classification and assessment	1,019 children from the CAMP study and 669 children from the ACRN/CARE dataset	PP	Clinical	Pediatric asthma phenotypes	Four phenotypes: allergic-not-obese, obese-not-allergic, allergic-and-obese, and not-obese-not-allergic	Pros: discovery of more detailed predictive features for long-term asthma control other than the current control state Cons: elimination of some features due to missing data
Wu W, 2019 46	Classification and assessment	346 adult asthma in the Severe Asthma Research Program	Multiple-kernel k-means	Clinical, physiological, inflammatory, demographic	Asthma control state	Four phenotypes: clusters 1 and 2: young modestly corticosteroid responsive allergic asthmatics with relatively normal lung function; cluster 3: late onset asthmatics with low lung function; cluster 4: primarily young obese females with severe airflow limitation	Pros: identification of phenotypes based on corticosteroid responses Cons: limited to a single dose of systemic corticosteroid (without placebo) and a single point in time
Prosperi MC, 2014 47	Classification and assessment	554 asthma adults	LR, RF, DT, AB	Clinical, genetic	Current asthma, wheeze, eczema	Optimal AUC = 0.84, 0.76 and 0.64 for asthma, wheeze, and eczema, respectively	Pros: integrated genomics information Cons: genetic analysis was restricted to candidate genes
Krautenbacher N, 2019 48	Classification and assessment	260 individuals: healthy children = 43%, mild-to-moderate, allergic asthmatics = 47%, nonallergic asthmatics = 11%	Lasso regression, elastic net, RF	Genetic, immunological, environmental	Asthma phenotypes	AUC for three classes of phenotypes = 0.81	Pros: identification of three important genes for classifying childhood asthma phenotypes: PKN2, PTK2 and ALPP Cons: should be validated in other cohort studies
Williams-De Vane CR, 2013 49	Classification and assessment	205 individuals	DT	Clinical, genetic, demographic	Asthma endotypes	Decision tree-based methods were useful tools for identifying asthma endotypes	Pros: integrated data to identify asthma endotypes Cons: should be validated in external data
Siroux V, 2014 50	Classification and assessment	3,001 asthmatic adults	LCA	Clinical (questionnaire data), genetic	Asthma phenotypes	Four phenotypes: inactive/mild nonallergic asthma, inactive/mild allergic asthma, active allergic asthma, and active adult-onset nonallergic asthma	Pros: large sample of asthmatic adults Cons: lack of formal replication of the genetic association signals
Mäkikyrö EM, 2017 51	Classification and assessment	1,995 asthma subjects	LCA	Clinical (questionnaire data), asthma-related healthcare use	Asthma phenotypes	Four subtypes for women: mild asthma, moderate asthma, unknown severity, and severe asthma. Three subtypes for men: mild asthma, unknown severity, and severe asthma.	Pros: development of a simpler way to categorize asthmatic subtypes Cons: did not test the population for biomarkers and form endotypes; did not verify the subtypes with full scale lung function testing
Nabi FG, 2019 52	Classification and assessment	55 asthma patients	Ensemble, SVM, KNN	Wheeze sounds	Asthma severity	The best positive predictive value for the mild, moderate, and severe samples were 95% (ensemble), 88% (ensemble) and 90% (SVM), respectively.	Pros: classified wheeze sounds of asthmatic patients according to severity Cons: relatively small sample
Moustris KP, 2012 53	Management and monitoring	3,602 children	ANN	Meteorological and ambient air pollution data	Childhood asthma admissions	Index of Agreement = 0.837 Coefficient of determination = 0.528	Pros: predicted the childhood asthma admission based on the bioclimatic and air pollution Cons: some environmental factors had not been included, such as relative humidity
Messinger AI, 2019 54	Management and monitoring	128 asthmatic children: training set = 102 testing set = 26	ANN	Demographic, clinical (EHR)	Respiratory score	The performance of pediatric-automated asthma severity scores was better than Pediatric Asthma Score.	Pros: pARS had the potential to help standardize acute pediatric asthma care in the PICU. Cons: incomplete data from the clinical record and sign database; a single center study
Xiang Y, 2020 55	Management and monitoring	31,433 adult asthma patients	ANN	Clinical (EHR)	Asthma exacerbation	AUC = 0.7003	Pros: a time-sensitive predictive model Cons: some potential risk factors for asthma exacerbations might not be recorded or might even be incorrectly recorded
Khatri KL, 2018 56	Management and monitoring	Patients of visiting emergency departments in Dallas County for respiratory diseases	ANN	Clinical, meteorological and environmental pollution data	Emergency department visits	Overall accuracy = 81.0%	Pros: can serve as useful tool for peak demand prediction in emergency departments Cons: limited number of variables; primary diagnosis may not be accurate
Grunwell JR, 2020 57	Management and monitoring	513 asthmatic children	LCA	Clinical, demographics	Asthma exacerbation	The class of multiple sensitizations with partially reversible airflow limitation had the highest exacerbation risk (64.3%)	Pros: prediction of exacerbation in school-age children Cons: factors responsible for asthma exacerbations were not adequately addressed by the study design
Fitzpatrick AM, 2020 58	Management and monitoring	2,593 children with mild to moderate asthma aged 5-18 years	LCA	Clinical, demographics, lung function test	Lung function and exacerbation rate	Children who had multiple sensitizations with partially reversible airflow limitation had the highest exacerbation risk (52.5%)	Pros: large sample size of diverse and representative children across the United States Cons: model selection for LCA can be subjective
Das LT, 2017 59	Management and monitoring	2,691 asthmatic children	LR, Lasso regression, RF, SVM	Clinical (EHR)	Emergency department visits	AUC = 0.86 reached by LR	Pros: based on electronic health records (EHRs) Cons: record of emergency department visits to one medical center
Zhang O, 2020 60	Management and monitoring	2,010 asthma patients	LR, DT, NB, perceptron algorithms	Daily monitoring data	Asthma exacerbations	AUC = 0.85, sensitivity = 90%, and specificity = 83% reached by LR	Pros: use of a large international dataset to detect severe asthma exacerbations Cons: data were collected using paper diaries, which be inaccurate or fabricated
Luo L, 2018 61	Management and monitoring	6,813 admission records	XGBoost	Search index, air pollution data, weather data, historical admissions	Asthma admission	AUC = 0.832	Pros: use of an easily accessible and daily updated daily search index Cons: data from a single geographical region
Ram S, 2015 62	Management and monitoring	Emergency department visits for asthma to the Children's Medical Center of Dallas (between October 2013 and December 2013)	ANN	Twitter data, Google search interests, environmental data	Emergency department visits	Accuracy = 70%	Pros: based on real-time environmental and internet-based data Cons: data of emergency department visits from one hospital
Finkelstein J, 2016 64	Management and monitoring	7,001 records submitted by adult asthma patients	NB, BN, SVM	Daily self-monitoring reports	Asthma exacerbations	BN model reached sensitivity, specificity, and accuracy of 100%	Pros: use of home telemonitoring data Cons: the number of cases of asthma exacerbations was small
Huffaker MF, 2018 65	Management and monitoring	33 subjects	RF	Recorded physiologic data	The time period during which onset of asthma symptoms occurred	Sensitivity = 47.2%, specificity = 96.3%, accuracy = 87.4%	Pros: showed that passive physiologic monitoring can be used in the home to assess asthma control Cons: small sample
Luo L, 2020 66	Management and monitoring	Cost data of asthmatic patients	LR, RF, SVM, classification regression tree, backpropagation neural network	Cost data	Treatment cost	AUC and sensitivity increase of 46.89% and 101.07%, respectively	Pros: use of machine learning to predict high cost Cons: lack of analysis of low-frequency comorbidities
Khasha R, 2019 67	Management and monitoring	96 asthma patients	LR, XGBoost, RF, DT, KNN, NB, SVM	Clinical, demographics, lung function test	Control level	Optimal accuracy = 91.66%	Pros: developed a novel ensemble learning method for asthma control level detection Cons: limited factors affecting asthma control were included
Tsang K, 2020 68	Management and monitoring	5,875 asthma patients	LR, NB, DT, SVM	mHealth data	Stable and unstable periods	Optimal sensitivity = 86.6%, optimal specificity = 72.5%, optimal AUC = 0.871	Pros: personalized algorithms to enhance asthma management Cons: self-reported data rather than objective measures
Hosseini SA, 2020 69	Treatment	80 patients with mild or moderate allergic asthma	ANN	Clinical, immunologic, hematologic, demographic	Low to high level of effect	Accuracy>99%	Pros: new machine learning model for the prediction of asthmatic drug effectiveness Cons: relatively small sample

**Abbreviations:** AB, AdaBoost; ADAB, AdaBoost with decision trees; AERD, aspirin-exacerbated respiratory disease; ANN, artificial neural networks; AUC, area under the receiver operating characteristic curve; BN, Bayesian networks; BIC, Bayesian Information Criterion; DT, decision trees; DNN, deep neural network; EMRs, electronic medical records; EHR, electronic health records; FDSC, feature-based dissimilarity space classifier; KNN, k-nearest neighbor; LCA, latent class analysis; LR, logistic regression; NB, naïve Bayesian; NLP, natural language processing; PP, predictor pursuit; RF, random forest; SVM, support vector machine.

**Table 3:** Machine learning studies on chronic obstructive pulmonary disease

Reference	Category	Study population	ML algorithms	Input features	Studied outcome	Results	Critical appraisal of the study
Matsumura K, 2020 74	Screening and diagnosis	non-smokers 68 smokers = 88 COPD subjects = 48	RF	Genetic (transcriptomic data)	Smokers or early stage of COPD	Each group with 65% accuracy. The discrimination accuracy of COPD subjects from smokers was only 29%	Pros: identification of novel genes associated with COPD Cons: limited number of patients with clear description of the smoking status
Zheng H, 2020 75	Screening and diagnosis	COPD patients = 54 normal individuals = 74	SVM	Serum metabolic biomarkers	COPD subjects or not	Accuracy = 84.62%, AUC = 0.90	Pros: based on serum metabolomics Cons: should be validated in a larger clinical sample
Haider NS, 2020 78	Screening and diagnosis	COPD patients = 30 healthy subjects = 25	SVM, KNN, LR, DT, DA	Clinical (lung sound), spirometry features	COPD subjects or not	Optimal accuracy = 100%	Pros: combination of spirometry data with lung sound features for COPD diagnosis Cons: small sample and single-center data
Spathis D, 2019 79	Screening and diagnosis	132 patients	NB, LR, ANN, SVM, KNN, DT, RF	Clinical, demographic	Asthma or COPD	Optimal accuracy = 97.7%	Pros: identification of COPD based on 22 different clinical features Cons: relatively small sample
Al Sallakh MA, 2018 82	Screening and diagnosis	Secure Anonymised Information Linkage (SAIL) Databank	LCA	Clinical (EHR)	Asthma-COPD overlap	A protocol	Pros: based on electronic health records (EHRs) Cons: incomplete information of electronic health records
Pikoula M, 2019 84	Classification and assessment	30,961 COPD patients	K-means, hierarchical clustering	Clinical (EHR)	COPD phenotypes	Five phenotypes: anxiety/depression; non-comorbid; cardiovascular/diabetes; severe COPD/frailty; obesity/atopy	Pros: identification of phenotypes based on EHRs Cons: unclear boundaries of some clusters
Burgel PR, 2017 85	Classification and assessment	6,060 COPD patients	CART	Clinical	COPD phenotypes	Five phenotypes: mild respiratory, moderate-to-severe respiratory, moderate-to-severe comorbid/obese, very severe respiratory, very severe comorbid	Pros: integrated respiratory characteristics and comorbidities Cons: assessment of comorbidities was based on physician diagnoses that did not consider occult conditions
Yoon HY, 2019 86	Classification and assessment	1,195 COPD patients	K-means	Clinical (seven variables)	COPD phenotypes	Four phenotypes: putative asthma-COPD overlap, mild COPD, moderate COPD, severe COPD	Pros: demonstrated that phenotype is linked to the occurrence of acute exacerbation Cons: short follow-up duration
Kim WJ, 2018 87	Classification and assessment	1,676 COPD patients from 13 Asian cities	Hierarchical cluster analysis	Clinical	COPD phenotypes	Three phenotypes: worse lung function and fewer symptoms, worse lung function and more symptoms. milder COPD and a preserved FEV1 and FEV1/FVC ratio	Pros: identification of COPD subgroups in a large Asian sample Cons: 90% male subjects
Castaldi PJ, 2014 88	Classification and assessment	10,192 smokers	K-means	Clinical	COPD phenotypes	Four phenotypes: relatively resistant smokers, mild upper zone emphysema-predominant, airway disease-predominant, severe emphysema	Pros: identification of phenotypes based on airway disease and emphysema Cons: non-inclusion of biomarkers and comorbidities
Bodduluri S, 2020 90	Classification and assessment	8980 individuals	DNN, RF	Spirometry data	Chest CT phenotypes (normal, airway predominant, emphysema predominant, and mixed emphysema/airway)	The DNN model had the highest accuracy (AUC = 0.80 and 0.91)	Pros: used spirometry data to train the model Cons: nonsmokers with and at risk for COPD were not included in the cohort
Gawlitza J, 2019 94	Classification and assessment	75 COPD patients	KNN, XGBoost, ANN	Quantified computed tomography	Pulmonary function	KNN model with the lowest mean relative error (16%)	Pros: prediction of lung function values from quantitative computed tomography parameters Cons: small sample
Westcott A, 2019 95	Classification and assessment	95 COPD patients	LR, SVM	Thoracic computed tomography	Lung ventilation	Accuracy = 88%, AUC = 0.82	Pros: development of a computed tomography analysis pipeline Cons: few mild COPD patients
González G, 2018 96	Classification and assessment	8,983 COPDGene participants and 1,672 ECLIPSE participants	Convolutional neural network	Chest computed tomography	COPD, stage, acute respiratory disease events, mortality	C-index = 0.856, accuracy = 51.1% in COPDGene cohort	Pros: based on chest computed tomography images Cons: high training computational cost and memory requirements
Peng J, 2020 97	Classification and assessment	410 hospitalized AECOPD patients	DT	Clinical (medical records)	Mild and severe AECOPD	Accuracy = 80.3%	Pros: fast identification of the deterioration and death risk of AECOPD patients Cons: non-inclusion of interleukin and other inflammatory cytokines
Goto T, 2019 98	Management and monitoring	44,929 hospitalized COPD patients	Lasso regression, DNN	Clinical	30-day readmission	C-statistic = 0.61	Pros: huge sample size and more than 1000 predictors Cons: unable to identify patients readmitted to different hospitals
Min X, 2019 99	Management and monitoring	111,992 patients from the Geisinger Health System	LR, RF, SVM, GBDT, MLP	Medical claims data	30-day readmission	Optimal AUC = 0.653	Pros: combined knowledge and data driven features Cons: lack of mortality information for patients
Cavailles A, 2020 100	Management and monitoring	143,006 patients hospitalized for AECOPD	DT	Clinical	Risk of readmission	Previous admission times was the most important risk of readmission	Pros: identification of variables associated with readmission Cons: no information on spirometry or severity
Chen W, 2020 101	Management and monitoring	4,167 subjects	RF	Clinical, spirometry	Prebronchodilator FEV1, risk of airflow limitation	C-statistic = 0.86-0.87	Pros: development of a personalized risk model to predict the risk of airflow limitation Cons: lack of ethnic diversity in the cohort
Ma X, 2020 102	Management and monitoring	COPD patients = 441 control subjects = 192	KNN, LR, DT, SVM, ANN, XGBoost	Genetic, clinical	Early-stage COPD	KNN and LR had the highest precision (82%) and accuracy (81%) ANN had the highest sensitivity (100%)	Pros: identification of the association of genes and COPD development Cons: unbalanced samples from the seven centers; only nine 9 genes and five clinical features were obtained
Lanclus M, 2019 103	Management and monitoring	62 COPD patients	SVM	Functional respiratory imaging	COPD exacerbations	Accuracy = 80.65%, positive predictive value = 82.35%	Pros: use of functional respiratory imaging for AECOPD prediction Cons: more advanced COPD patients in the cohort
Wang C, 2020 104	Management and monitoring	AECOPD patients = 135 not AECOPD patients = 168	RF, SVM, LR, KNN, NB	Clinical (EMRs)	COPD acute exacerbations	Optimal sensitivity = 80%, specificity = 83%, positive predictive value = 81%, negative predictive value = 85%, and AUC = 0.90 from SVM	Pros: decision support for clinicians Cons: single-center cohort
Luo L, 2020 107	Management and monitoring	780,295 hospitalizations data	LR, RF, XGBoost	Medical insurance data	High-cost COPD patients	AUC = 0.787 (LR); AUC = 0.792 (RF); AUC = 0.801 (XGBoost)	Pros: identification of high costs for COPD patients Cons: no smoking status, household income, or family population information
Morales DR, 2018 108	Management and monitoring	54879 COPD patients	LR, SVM	Clinical	1-year mortality	C-statistic = 0.723	Pros: use of external data to validate models Cons: analysis of patients only with complete data
Moll M, 2020 109	Management and monitoring	2,632 participants from COPDGene cohort and 1,268 participants from ECLIPSE cohort	RF	Clinical, spirometry, imaging	Time to death from any cause	C-index ≥ 0.7 in both cohorts	Pros: prediction of all-cause mortality Cons: cohorts were not representative of the general population
Orchard P, 2018 110	Treatment	135 COPD patients	Sparse maximum-margin classifier, ensembles of boosted classifier, multitask neural network model	Clinical (telemonitoring data), weather	Admission and initiation of oral corticosteroid treatment	Optimal AUC = 0.74	Pros: the model serves as a guide for corticosteroid therapy Cons: lack of a gold standard definition for exacerbation

**Abbreviations:** ANN, artificial neural networks; AUC, the area under the curve; BN, bayesian networks; CART, classification and regression tree; DA, discriminant analysis; DNN, deep neural network; DT, decision trees; EMRs, electronic medical records; EHR, electronic health records; GBDT, gradient boosting decision tree; KNN, k-nearest neighbors; LCA, latent class analysis; LR, logistic regression; MLP, multi-layer perceptron; NB, naïve Bayes; RF-random forest; SVM-support vector machine.

## Abbreviations

ACO: asthma-COPD overlap
AECOPD: acute exacerbations of COPD
AI: artificial intelligence
ML: machine learning
AUC: area under the receiver operating characteristic curve
COPD: chronic obstructive pulmonary disease
ECLIPSE Study: “Evaluations of COPD longitudinally to identify predictive surrogate endpoints”
EHRs: electronic health records
FEV1: forced expiratory volume in 1 second
